# Understanding the State of US-Based Spanish Language Wildfire Outreach and Education Materials for the Public: A Case Study of California

**DOI:** 10.1007/s00267-025-02361-5

**Published:** 2026-01-30

**Authors:** Samrajya Bikram Thapa, Jeanette Cobian-Iñiguez

**Affiliations:** University of California, Davis, California USA

**Keywords:** Risk communication, Wildfire preparedness, Educational materials, Geographic disparities, Language barriers, Underserve communities

## Abstract

Wildfires pose an escalating threat to communities across California, with Spanish-speaking populations facing disproportionate vulnerabilities due to limited access to culturally and linguistically appropriate educational resources. This study examines the availability, accessibility, and spatial distribution of Spanish-language wildfire education materials across the state. We combined quantitative trend analysis with qualitative content and cluster analyses to identify key content gaps and geographic disparities. Results reveal that while the production of Spanish-language materials has increased in recent years, it remains inconsistent and significantly lags behind the availability of English-language resources. Outreach efforts are concentrated in wildfire-prone regions like Southern California, whereas other high-risk and socioeconomically disadvantaged regions, such as the Central Valley and parts of Northern California, are underserved. Thematic analysis of material content shows varying emphasis on preparedness, evacuation, and recovery, but limited coverage of long-term resilience and environmental justice concerns. Local agencies and non-profits have emerged as pivotal actors in addressing these gaps, yet their efforts are constrained by limited resources and coordination challenges. Systemic disparities in outreach stem from perceptions of audience demand and inconsistent funding mechanisms. Our findings underscore the urgent need for coordinated action across federal, state, and local agencies to ensure equitable dissemination of wildfire information. Strengthening collaborations and increasing investment in culturally relevant, Spanish-language materials will be essential for enhancing community resilience, improving wildfire preparedness, and closing critical communication gaps for at-risk populations. This study highlights actionable pathways for more inclusive and effective wildfire communication strategies across linguistically diverse regions of California.

## Introduction

Wildfires have become increasingly destructive in the western U.S., particularly in California, where climate change and urban expansion into fire-prone landscapes have escalated the frequency and severity of fire events (Radeloff et al. 2005; Abatzoglou and Williams 2016; Westerling 2016). The confluence of climate-driven aridity and rapid development in the wildland-urban interface (WUI) has not only increased fire risk (Syphard et al. 2017; Keeley and Syphard 2018), but also exposed critical gaps in inclusive disaster preparedness and communication strategies. One of the most pressing concerns is the lack of accessible wildfire education materials in languages other than English, particularly Spanish. This disparity has far-reaching implications—not only for public awareness—but also for wildfire response effectiveness, community resilience, and long-term risk mitigation.

California is home to the largest Hispanic and Latino population in the U.S., comprising ~39% of the state’s residents (U.S. Census Bureau 2020). Many Hispanic communities reside in fire-prone areas such as the Central Valley and Southern California (McGee and Russell 2004). These communities often face compounded vulnerabilities due to socioeconomic barriers, including lower income levels, limited formal education, and reduced access to healthcare—all of which can restrict their capacity to prepare for, respond to, and recover from wildfire events (Collins and Bolin 2009). Language barriers exacerbate these vulnerabilities by impeding access to timely, accurate, and actionable information.

Effective risk communication is foundational to wildfire response and resilience. Timely, understandable warnings can directly influence evacuation behavior and emergency decision-making (Lindell and Perry 2012; McCaffrey and Olsen 2012). However, when disaster messaging is primarily disseminated in English—as is often the case in California—non-English-speaking residents are left at a disadvantage. Studies show that individuals are significantly less likely to evacuate or follow emergency instructions when information is not available in their primary language (Perry and Lindell 2003; Huang et al. 2012; Morss et al. 2011). In high-intensity wildfire events, even minor delays in evacuation can lead to severe consequences, including loss of life (Steelman and McCaffrey 2013). Thus, the lack of Spanish-language materials directly affects response outcomes, increasing the risk of harm among already vulnerable groups.

The issue is not merely one of translation but of cultural competence. Risk communication must be culturally resonant to be effective. Research indicates that Hispanic communities often rely on collective decision-making and prioritize family-centered preparedness (Cutter and Finch 2008). Communication strategies that align with these values—through culturally adapted materials—are more likely to inspire action and foster resilience (Remenick 2017). Transcreation, or the process of adapting content for cultural and linguistic relevance, has proven effective in both health and disaster communication (Paveglio et al. 2018; Mockrin et al. 2023). In the context of wildfires, culturally resonant messaging can support not only individual behavior but also community-level preparedness and mutual aid, both of which are critical to resilient recovery (Cutter et al. 2014). The disparities in language-accessible materials also hinder risk mitigation efforts. Community preparedness is a key component of mitigation strategies, helping to reduce structural damage, improve evacuation plans, and build long-term adaptive capacity (Lachlan et al. 2010; Vaughan and Tinker 2009). However, limited Spanish-language outreach undermines these goals. Méndez et al. (2020) found that while some California agencies have developed Spanish-language materials, efforts remain inconsistent and lack strategic distribution across regions most at risk. Compared to hurricane-prone regions like Florida and Texas—where agencies has made notable progress in multilingual outreach (FCC; USFA 2021)—California lags in developing and disseminating culturally relevant wildfire preparedness resources (Baker et al. 2024; Hopfer et al. 2024). The Hispanic Access Foundation (2023) has noted a long-standing neglect of Spanish-speaking communities in emergency planning, contributing to informational disparities that ultimately impede mitigation effectiveness.

Furthermore, disparities in communication lead to misinformation and confusion during crises. In the absence of clear, official information in their primary language, Spanish-speaking residents may turn to informal networks, hearsay, or social media, where messages are not always verified (Mileti and Sorenson 1990; Vien et al. 2024). These gaps in formal communication channels can erode trust in public agencies, reduce compliance with safety protocols, and complicate emergency management operations. For fire agencies, this means not only delayed evacuations but also strained response resources and logistical inefficiencies during critical operations.

Systemic barriers have contributed to the uneven availability of Spanish-language wildfire materials. Historically, wildfire education programs were developed for predominantly English-speaking populations and did not account for California’s demographic evolution (van Wagtendonk 2007). Resource constraints remain a persistent issue; many public agencies and nonprofits lack the funding to employ bilingual staff, commission high-quality translations, or produce enough materials to reach all at-risk communities (O’Brien and Federici 2019; Masri et al. 2021). Moreover, the absence of coordination between state and local agencies often leads to fragmented outreach—where some communities receive duplicated information while others receive none (Steelman and McCaffrey 2013; Nowell and Steelman 2014).

Providing bilingual wildfire risk information is not only a matter of equity but also public safety. Research shows that people are more likely to understand and act on disaster information presented in their primary language, improving preparedness outcomes (Vaughan and Tinker 2009; Huang et al. 2012). In wildfire emergencies, where quick response is critical, bilingual materials can save lives and also help reduce misinformation (Huang et al. 2012). Vien et al. (2024) highlight ongoing shortfalls in Spanish-language wildfire smoke communication, and GreenLatinos emphasize the risks these gaps pose for Hispanic communities. Ensuring timely, accurate, and culturally relevant information in both English and Spanish can reduce confusion and support safer outcomes for all (Cutter et al. 2003).

Given the increasing frequency and intensity of wildfires in California and the growing recognition of the vulnerabilities faced by Hispanic and Latino communities, critical gaps remain in understanding the availability and accessibility of Spanish-language wildfire educational materials. The novelty of this study lies in its comprehensive examination of Spanish-language wildfire educational materials, focusing specifically on temporal trends and spatial disparities in their dissemination across California. Unlike previous research that broadly highlights the lack of multilingual risk communication, this study uniquely investigates the evolution of wildfire educational materials over time and their accessibility to non-English-speaking communities, particularly Spanish-speaking populations. Furthermore, it introduces a spatial variation analysis, revealing geographic disparities in the availability of these materials across regions with high concentrations of vulnerable populations. By addressing these overlooked areas, the study provides critical insights for developing more inclusive and effective wildfire communication strategies.

This study is guided by four objectives, each accompanied by a testable hypothesis. The first objective is to analyze temporal trends in the availability of Spanish-language wildfire educational materials in California. It is hypothesized that the proportion of Spanish-language materials remains significantly lower than that of English-language materials. The second objective seeks to identify spatial disparities in the distribution of Spanish-language wildfire materials across the state. It is hypothesized that regions with higher percentages of Spanish-speaking residents are not necessarily better resourced in terms of Spanish-language educational materials. The third objective explores the relationship between the type of agency producing materials and the availability of Spanish-language content. It is hypothesized that state and federal agencies are more prolific in producing Spanish-language materials than local or non-profit organizations. The final objective focuses on the thematic alignment between existing educational materials and the actual needs of Spanish-speaking communities. It is hypothesized that most Spanish-language wildfire materials emphasize general preparedness, with relatively limited attention to topics such as smoke exposure, evacuation, and post-disaster recovery.

## Methods

### Data Collection

This study used a systematic approach to collect wildfire education and outreach materials relevant to California, focusing on both English and Spanish content. The goal was to compile a dataset that supports both qualitative and quantitative analysis, including cataloging existing materials, translating Spanish-language resources, building a metadata database, and incorporating wildfire risk and demographic data for spatial analysis.

The first phase involved identifying and cataloging wildfire education materials from local, state, federal agencies, non-profits, and private sources following frameworks used in prior wildfire communication research that distinguish between institutional roles and outreach responsibilities across organizational levels (Steelman and McCaffrey 2013; McCaffrey 2015). Materials included brochures, flyers, posters, reports, and articles in both English and Spanish. Inclusion criteria required relevance to wildfire risk, a California focus, and language accessibility. Only text-based and print materials were included in the analysis. Multimedia formats such as videos, radio public service announcements were excluded due to inconsistent availability, metadata structure, and difficulty in systematic cataloging. A systematic search was conducted using predefined search terms in both English and Spanish to capture a broad array of documents. In Spanish, key search terms included “riesgo de humo de incendios forestales,” “prevención de incendios forestales,” “mitigación del riesgo de incendios forestales,” “prácticas de manejo,” “plan de evacuación,” “materiales sobre quemas prescritas,” and “guías de preparación para incendios forestales.” Corresponding English terms were also used to identify English-language materials. The search was conducted across multiple databases, ensuring the inclusion of widely disseminated and specialized materials. Relevant materials were sourced from the official websites of governmental organizations such as the California Department of Forestry and Fire Protection (CAL FIRE), the Federal Emergency Management Agency (FEMA), and the US Forest Service, as well as NGOs focused on fire preparedness and environmental outreach. Google search was used for general web resources and organizational publications.

### Translation of Spanish-Language Materials

Spanish-language documents were translated into English to ensure consistency in analysis. While AI tools aided preliminary content understanding, they were not used for final translation due to limitations in accuracy and cultural nuance (Naveen and Trojovsky 2024). Bilingual undergraduate translators reviewed all content to ensure fidelity in meaning, terminology, and tone. These translations ensured comparability across documents for both qualitative coding and quantitative assessment.

### Creating a Database

All materials were organized into a structured metadata database. This facilitated document classification, retrieval, and systematic analysis based on variables such as language, source, type, and content focus (Table 1). To support spatial analysis, two key datasets were integrated: wildfire risk data from the U.S. Forest Service (ArcGIS Online) and Spanish-speaking population data from the 2020 U.S. Census. USFS wildfire risk to communities dataset was selected because it provide nationally consistent and publicly available assessment of wildfire risk across all land types in the United States. It combined multiple components – burn probability, modeled fire intensity, exposure, and potential loss to homes into an integrated measure of risk. These layers enabled analysis of whether Spanish-language materials aligned with areas of high fire risk and large Spanish-speaking populations, identifying gaps in outreach coverage.

**Table 1:** Showing metadata for a database

Field	Description
1. File name	The exact name of the file, allowing for easy identification and retrieval.
2. Publication date	The date when the material was published, crucial for assessing temporal trends in wildfire communication.
3. Organization name	The name of the organization that produced the material, providing insight into source and aiding analysis of organizational involvement.
4. Main subject	The primary focus of the material for thematic analysis.
5. Major themes	The broader categories or issues addressed by the document
• Community engagement and collaboration • Health impacts • Insurance and financial • Wildfire mitigation and safety • Wildfire preparedness and evacuation plan	Focuses on fostering community involvement and partnerships to address wildfire risk. Health related consequences of wildfire, including respiratory issues, mental health, and long-term wellness. Topics related to wildfire insurance coverage, financial assistance, and economic impacts of wildfires. Strategies and practices aimed at reducing wildfire risk and enhancing safety measures. Planning and readiness for potential wildfire events, including evacuation routes and safety protocols
6. Agency types	The type of organization responsible producing the material.
• Federal • Local agencies • Non-profit • State • Private	Materials published or supported by federal government agencies, like the U.S. Forest Service, FEMA, and others. Materials developed by city, county, or local entities. Non-profit organizations focused on wildfire education, prevention, and recovery. Materials endorsed by state government agencies such as Cal Fire or state environmental protection departments. Materials from various other entities, including private-public partnerships, and more.
7. Material types	The classification of document, such as brochures, flyers, posters, reports, articles or guides.
• Articles • Brochures • Flyers	Detailed analysis on wildfire-related topics, often published in journals or online platforms. Informational pamphlets providing concise guidance or information on wildfire preparedness, safety, and related topics. Brief, usually single-page documents designed to quickly convey essential wildfire information to the public.
• Guides • Posters • Reports	Comprehensive documents offering step-by-step instructions on various aspects of wildfire-related topics. Detailed document presenting findings, data or recommendations on wildfire management, often used for policy and planning purpose. Detailed documents presenting data, findings, assessments, or recommendation related to wildfire management, risk reduction, or community impact.
8. Engagement types	The level of engagement offered by the material – interactive, informational, or educational, representing how the material aims to engage its audience.
• Collaborative • Educational • Informational • Interactive	Materials that emphasize teamwork, partnership, and collective action in addressing wildfire challenges. focused on teaching or providing knowledge about wildfire risks, safety measures, and preparedness strategies. Documents that provide straightforward information or updates on wildfire conditions, risks, and responses. Materials that engage the audience actively, such as quizzes, simulations, or participatory exercises related to wildfire education.
9. Audience types	The intended audience for the material, including researchers, general public, homeowners, or policymakers.
• General audience • Homeowners • Policy makers • Researchers • Workers/Employees	Materials aimed at the public, with broad, accessible information suitable for all community members. Materials providing advice and guidelines for property owners on how to protect their homes from wildfires. Documents intended for government officials or others in decision-making roles, focusing on policy, regulations, and strategic planning related to wildfire. Materials designed for the academic or scientific community, often including detailed data, analysis, or research findings on wildfires. Information tailored for professionals working in wildfire-related fields, including safety guidelines, training materials, and operational protocols. Information tailored for professionals in wildfire-related roles—such as firefighters, forestry staff, utility workers, construction crews, or emergency responders.
10. Address	The source address of the document, where applicable, including the Zip Code for spatial analysis.

## Data Analysis

### Qualitative Content Analysis

A structured content analysis was conducted on translated materials to identify themes and patterns. Each document was reviewed and cataloged in metadata. A three-stage coding process – open, axial, and selective. Open coding involved a meticulous line-by-line examination of the text to identify distinct concepts and assign initial codes. In axial coding, related codes were grouped to form categories, linking concepts, and identifying relationships. Finally, selective coding was used to extract key themes such as wildfire prevention, evacuation, health impacts, and community preparedness (Bryman 2012; Corbin and Strauss 2008). Sub-themes were also developed to capture nuanced content. The coding framework allowed for organizing themes based on frequency and significance. This process ensured consistency across the dataset and formed a solid foundation for further analysis.

### Quantitative Data Analysis

This study employed a range of statistical techniques to examine temporal patterns, variable importance, and spatial distribution of wildfire-related educational materials in California. All analyses were conducted using R programming, incorporating packages such as “dplyr”, “ggplot2”, “randomForest”, “mgcv”, “Kendall”, “ca”, and “factoextra”.

Temporal trends were explored through time-series visualizations of material production by type, language, and agency involvement. These visualizations captured annual fluctuations from 2000 to 2024, stratified by language, material type, and organizational source. The Mann–Kendall test was applied to detect monotonic trends in material production over time, providing robustness against violations of linearity and normality.

To explore variable importance and key predictors of material distribution, a random forest model was implemented using the “randomForest()”. Model performance was validated using the out-of-bag (OOB) error rate, and the final model achieved an OOB accuracy of 84.6%, which remained below 18%, indicating good predictive accuracy. Given its non-parametric nature and robustness to multicollinearity, random forest was particularly well suited for this dataset. Variable importance was quantified using the “importance()” function, and the top 15 predictors were visualized using bar plots.

Linear regression and Generalized Additive Models (GAM) (Wood 2017) were used to model both linear and non-linear relationships, with model performance compared using Akaike Information Criterion (AIC) (Akaike 1974) to avoid overfitting and optimize predictive accuracy. Model assumptions were tested: residual normality was assessed with the Shapiro–Wilk test, homoscedasticity with the Breusch–Pagan test, and multicollinearity with Variance Inflation Factor (VIF); any variable with VIF > 5 was excluded. Model selection was performed using stepwise AIC, and models with lowest AIC score were retained.

To evaluate geographic disparities in the distribution of wildfire-related educational materials across California, a structured spatial analysis was conducted using the ZIP code-level metadata. Geographic coordinates were derived from ZIP codes using the zipcodeR package, then converted into spatial point features with the sf package. All spatial layers were projected to the California Teale Albers projection to ensure spatial consistency across data sources. Spatial overlay incorporated wildfire risk area data and percentage of Spanish-speaking populations at the Zip code level. The combined dataset was then analyzed using k-means clustering to classify regions based on standardized z-scores of wildfire risk, Spanish-speaking population percentage and total educational materials. The optimal number of clusters (*k* = 4) was determined by the elbow method using the fviz_nbclust() function based on within-cluster sum of squares. To assess the assumption of spatial independence, spatial autocorrelation was evaluated using Moran’s I statistic from the spdep package. In the pre-adjustment model, Moran’s I indicated significant spatial autocorrelation in material availability (*I* = 0.276, *p* < 0.001), suggesting clustering of similar values. To account for this, a spatial lag model “lagsarlm()” was fitted to evaluate spatially structured residuals. Additionally, ZIP codes identified as high leverage outliers were removed based on Cook’s distance and standardized residuals. Following these check, Moran’s I was recalculated on the adjusted input data, showing no significant spatial autocorrelation (*I* = 0.041, *p* = 0.154), confirming that clustering outputs were nor driven by spatial dependence. Each cluster was color-coded to represent regions with similar risk and demographic profiles, and the availability of wildfire educational materials was analyzed across these clusters. This clustering approach provided insights into regional disparities, allowing us to identify areas with similar demographic and risk profiles and assess whether educational resources were equitably distributed.

Finally, correspondence analysis was used to explore relationships between categorical variables (agency type, material theme, audience engagement), visualized using biplots. This multi-method approach provided a comprehensive understanding of the distribution and equity of wildfire communication efforts across the state.

## Results

### Trend Analysis

**Fig. 1:**
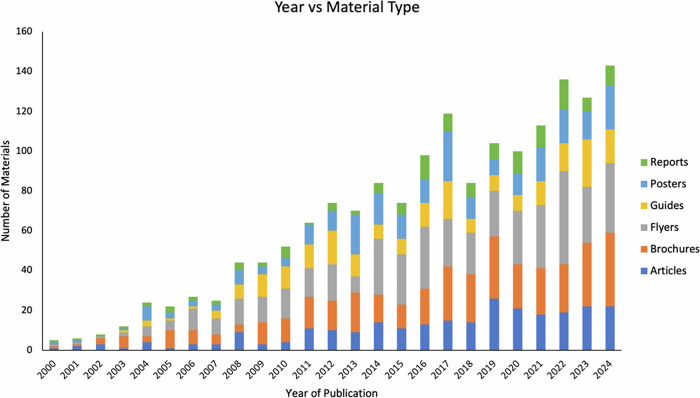
Temporal trends in wildfire education material types

**Fig. 2:**
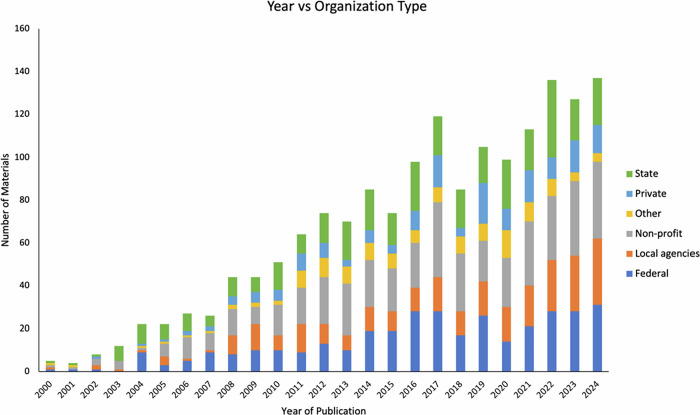
Temporal trends in organizational involvement in English wildfire educational material production

The trend analysis of wildfire educational materials in California reveals a clear growth in English-language resources, with material types showing distinct patterns (Fig. 1). Brochures and flyers fluctuate significantly, likely due to seasonal or event-driven outreach, while reports and guides show a steady increase, reflecting their foundational role. Different material types exhibit simultaneous highs and lows, suggesting resource reallocation or shifting strategies among agencies. The rise is supported by consistent contributions from state, non-profit, and federal agencies (Fig. 2).

**Fig. 3:**
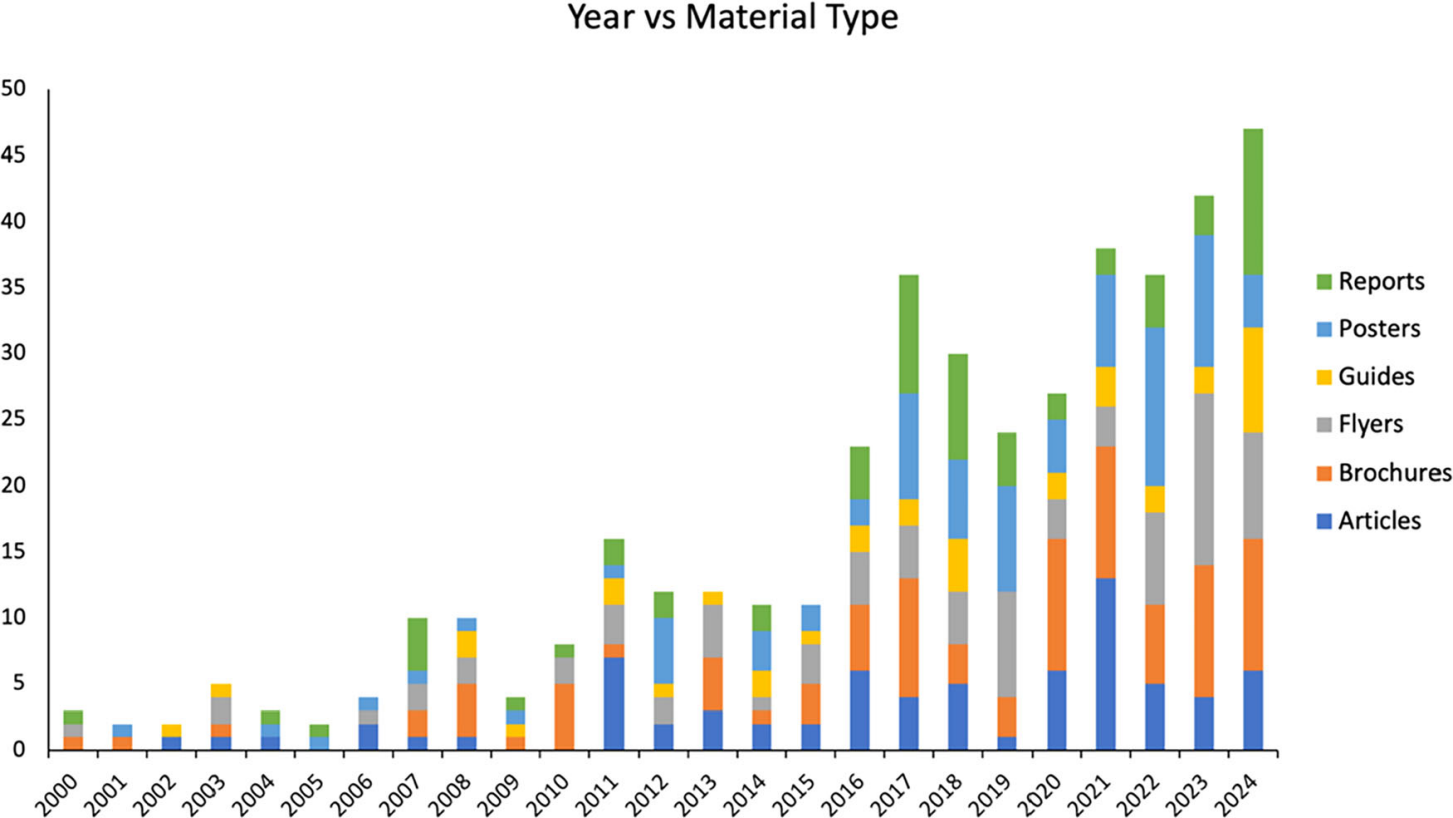
Temporal distribution of Spanish-language wildfire educational material types

**Fig. 4:**
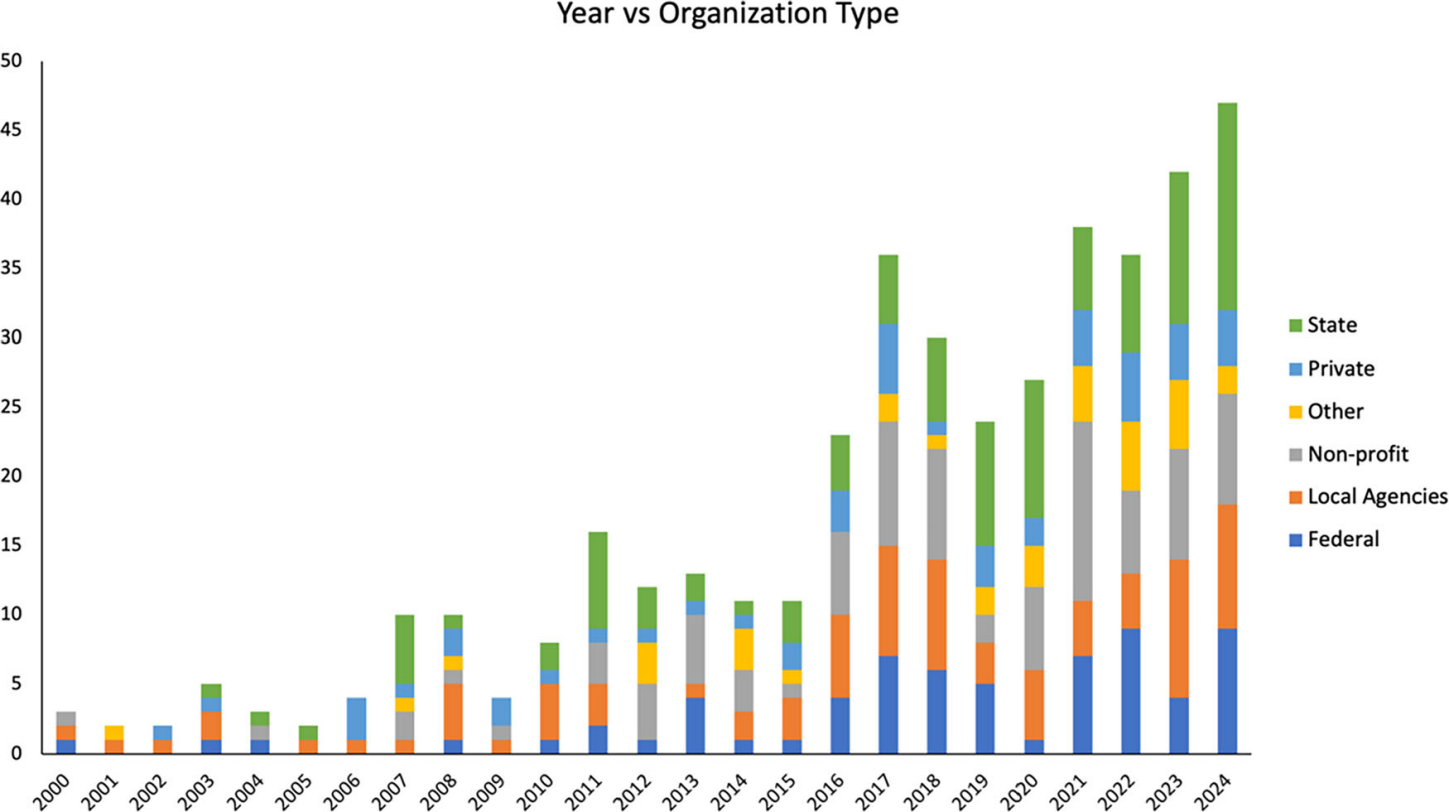
Temporal distribution of or organizational involvement in Spanish-language wildfire educational material production

In contrast, Spanish-language materials show a sporadic and inconsistent trend (Fig. 3), with lower and erratic publication rates. This pattern may reflect limited resources, unclear audience targeting, or low perceived demand. Involvement from agencies in producing Spanish materials is less consistent (Fig. 4). The trend analysis confirmed a significantly lower and inconsistent growth of Spanish-language materials compared to English-language resources, supporting the first hypothesis of continued disparity in availability. An analysis of “Material Types” categorized by bilingual experts shows that non-technical materials dominate (56%), followed by informative guides (19%) and technical materials (15%). From 2018 to 2021, non-technical materials steadily increased, highlighting a growing emphasis on accessible content in wildfire outreach.

### Variable Importance

**Fig. 5:**
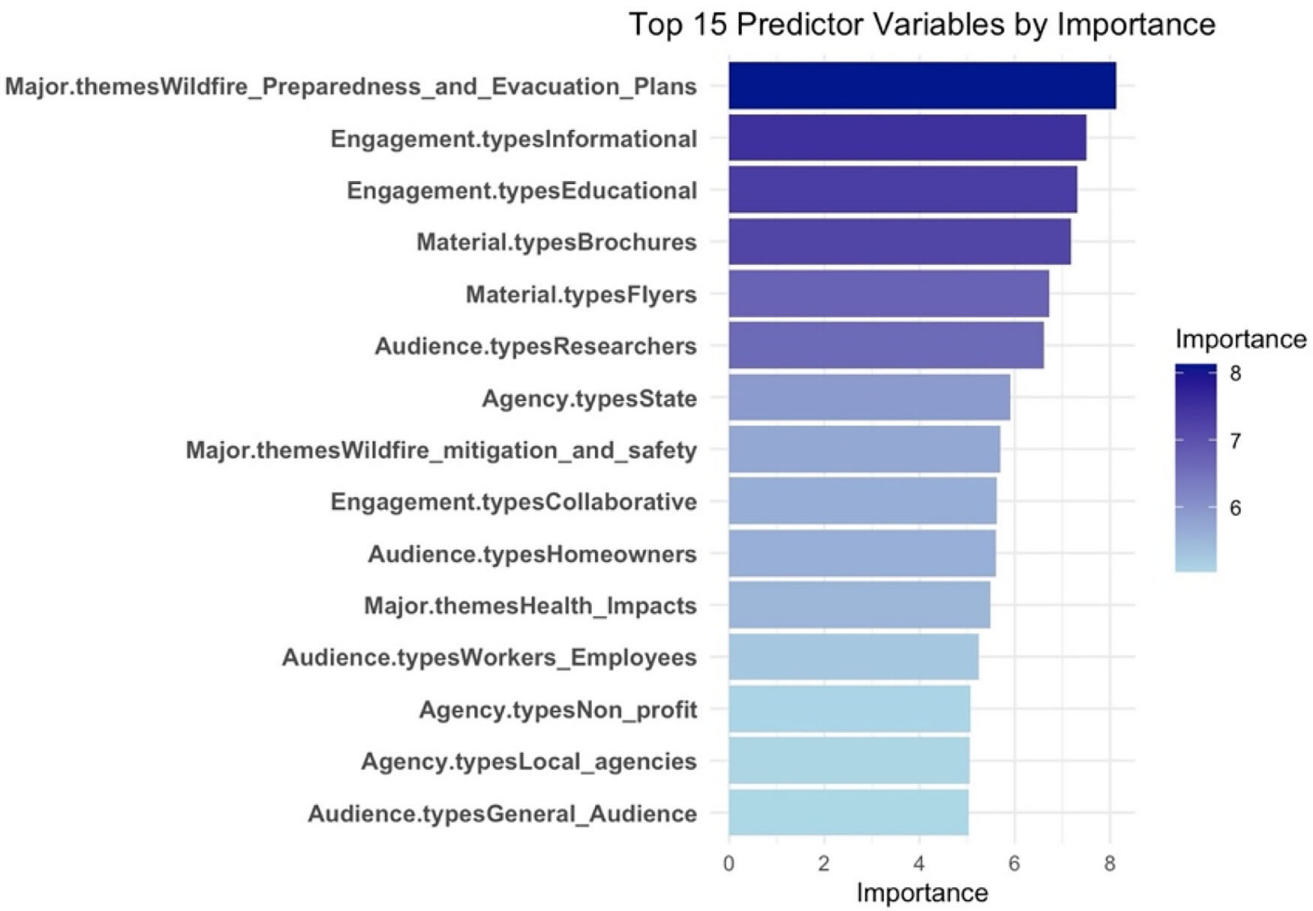
Top 15 predictor variables ranked by importance from the Random Forest Model

A random forest model identified important variables influencing the availability of wildfire educational materials (Fig. 5). “Wildfire Preparedness and Evacuation Plans” under “Major Themes” was the most important variable, followed by “Informational” and “Educational” engagement types. Brochures and flyers were key material formats, while state agencies ranked high in importance. Themes like “Wildfire Mitigation and Safety” and “Collaborative” engagement types were also influential. Less important variables included non-profit and local agencies, and audience types like “Homeowners”, “Workers/Employees”, and the “General Audience”.

### Regression Analysis

The trend analysis of wildfire-related educational materials in California, using both linear and GAM regression models along with Mann–Kendall tests, identifies significant growth across key areas (Table 2). Within “Major Themes,” *Health Impacts*, *Wildfire Mitigation and Safety*, and *Preparedness and Evacuation Plans* show highly significant trends (*p* < 0.01), with strong Mann–Kendall scores (0.839 for preparedness). In contrast, *Insurance and Financial Concerns* and *Community Engagement* show no significant increases. Model 2 consistently outperformed Model 1, with AIC reductions from 109.34 to 106.14 (linear) and 105.07 to 104.01 (GAM).

**Table 2:** Shows results of linear and non-linear (GAM) regression models for wildfire educational materials in California

			Linear				GAM			Mann–Kendall	
Groups	Variables	Model 1		Model 2		Model 1		Model 2		Score	Trend
		*p*-value	AIC	*p*-value	AIC	*p*-value	AIC	*p*-value	AIC		
Major themes	Community engagement and collaboration	0.45639	109.3421		106.1425	0.44811	105.0677		104.012	0.667	
	Health impacts	0.00270**		0.00222**		0.00463**		0.00315**		0.763	Positive
	Insurance and financial	0.84369				0.31296				0.491	
	Wildfire mitigation and safety	0.00913**		0.003499**		0.01548**		0.00550**		0.783	Positive
	Wildfire preparedness and evacuation plan	0.00791**		0.000448*		0.01258**		0.00297**		0.839	Positive
Agency	Federal	0.109294	114.2973		112.321	0.098608	110.6865	0.052315	106.0665	0.658	Positive
	Local agencies	0.019875**		0.013780**		0.018534**		0.000238**		0.743	Positive
	Non-profit	0.000652**		0.000417**		0.000387**		0.000005**		0.805	Positive
	Other	0.240438				0.402444				0.516	
	Private	0.897531				0.855198				0.624	
	State	0.209932				0.404535				0.721	Positive
Audience	General audience	0.1695	112.9936	0.0103*	111.3518	0.1681	111.6584	0.00986**	111.6071	0.822	Positive
	Homeowners	0.0343*		0.0343*		0.0191**		0.06009		0.809	Positive
	Policy Makers	0.4259				0.314				0.545	
	Researchers	0.0795				0.0140**		0.02262**		0.744	Positive
	Workers/employees	0.3545				0.2033				0.777	Positive
Material types	Articles	0.497355	107.1377		102.9769	0.266335	100.0372		98.57151	0.578	
	Brochures	0.000154**		0.00005**		0.000328**		0.000451**		0.858	Positive
	Flyers	0.001696**		0.00104**		0.000557**		0.000308**		0.794	Positive
	Guides	0.298793				0.270458				0.545	
	Posters	0.682354				0.338297				0.481	
	Reports	0.354629				0.589318		0.132212		0.61	
Message types	Collaborative	0.15593	113.2628		109.4555	0.07204	108.8422	0.05400*	106.7109	0.763	Positive
	Educational	0.00520*		0.002846*		0.00522**		0.00350**		0.808	Positive
	Informational	0.00795*		0.000186*		0.01068**		0.00211		0.825	Positive
	Interactive	0.94287				0.89328				0.619	
	Participatory	0.382				0.5698				0.69	

* significant at *p* < 0.05 ** significant at *p* < 0.01

Among agencies, *Non-profit* and *Local* entities showed the most significant growth (*p* < 0.001; Mann–Kendall = 0.805, 0.743), while federal and state agencies exhibited positive but less significant trends. AIC scores improved from 114.30 to 112.32 (linear) and 110.69 to 106.07 (GAM). For audiences, *Homeowners* and the *General Public* were the most targeted (*p* < 0.05; Mann–Kendall = 0.809), with Model 2 AIC dropping from 112.99 to 111.35 (linear) and 111.66 to 111.61 (GAM). Contrary to the third hypothesis, analyses indicated that non-profit and local agencies, rather than state or federal organizations, played a more prominent role in producing Spanish-language materials.

In “Material Types,” *Brochures* and *Flyers* showed the strongest growth (*p* < 0.001; Mann–Kendall = 0.858, 0.794), with AIC falling from 107.14 to 102.98 (linear) and 100.04 to 98.57 (GAM). *Educational* and *Informational* messages also increased significantly (*p* < 0.01), unlike *Collaborative* and *Participatory* types. Model 2 again performed better, reducing AIC from 113.26 to 109.46 (linear) and 108.84 to 106.71 (GAM). Overall, results highlight expanding efforts in preparedness, health-focused messaging, and non-profit/local outreach, with brochures and flyers as the dominant formats.

### Cluster Analysis

**Fig. 6:**
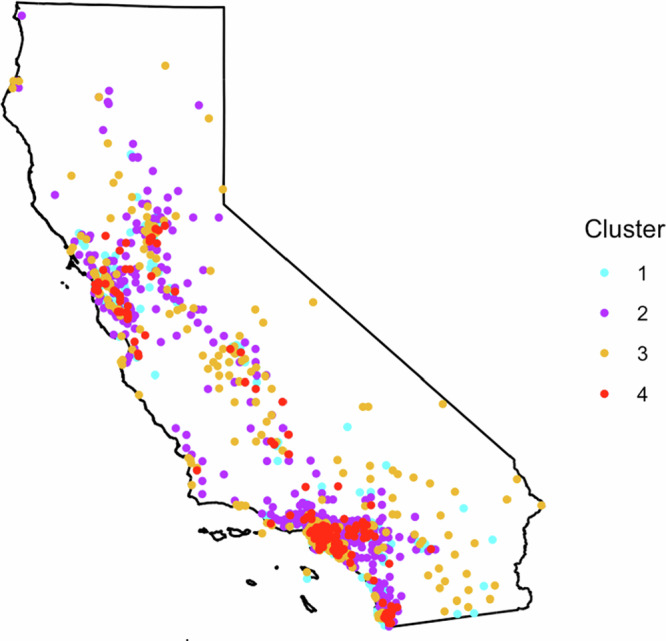
Spatial distribution of wildfire risk and educational outreach clusters across California. Four clusters from k-means analysis combine wildfire risk, Spanish-speaking population density, and material availability: (1) low-moderate risk, sparse Spanish populations, moderate materials; (2) moderate-high risk, high Spanish populations, limited materials; (3) moderate risk, high Spanish populations, low-moderate materials; and (4) very high risk, high Spanish populations, strong outreach

The cluster analysis (Fig. 6) identified four distinct spatial patterns across California based on wildfire risk, Spanish-speaking population density, and the availability of wildfire-related educational materials. Cluster 1 (cyan) comprises areas with low to moderate wildfire risk, sparse Spanish-speaking populations, and moderate availability of materials. Cluster 2 (purple), primarily located in Central and Southern California, includes regions with moderate to high wildfire risk, high Spanish-speaking populations but limited access to educational materials. Cluster 3 (golden-brown) represents areas with moderate wildfire risk, high Spanish-speaking populations and low to moderate material availability. Cluster 4 (red), concentrated in Southern California, is characterized by very high wildfire risk, dense Spanish-speaking populations, and substantial material availability. These clusters highlight considerable disparities in outreach, particularly in areas where high-risk and high-need populations are underserved.

**Fig. 7:**
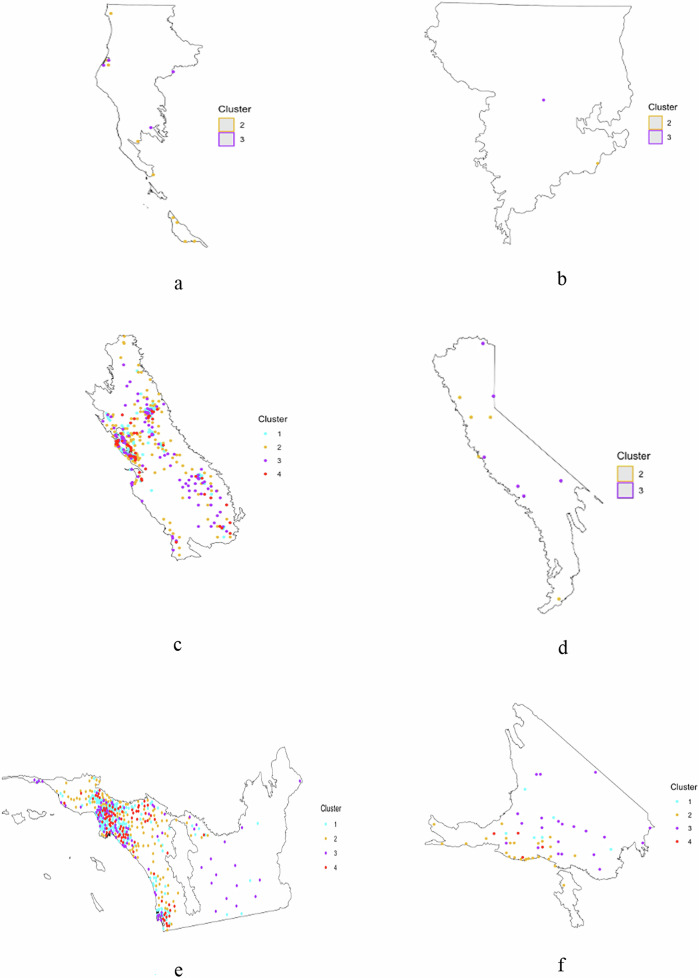
Presents series of maps illustrating the spatial distribution of clusters related to wildfire risk, Spanish-speaking population density, and the availability of wildfire-related educational materials across several ecoregions in California. Each map corresponds to a specific ecoregion: Outer Northern California (**a**), Inner Northern California (**b**), Central Valley and Central Coast (**c**), Sierra region (**d**), Southern California (**e**), and Southern California Mountain (**f**)

Further analysis of regional patterns (Table 3, Fig. 7a–f) underscores the variability of educational outreach across California’s ecoregions. In Southern California, the presence of all four clusters suggests that while high-risk, high-density areas are relatively well-covered, remote regions still face gaps in outreach. The Central Valley and Central Coast, despite moderate to high wildfire risk and significant Spanish-speaking populations, show limited material access, indicating the need for enhanced outreach efforts. In Outer Northern California, where wildfire risk is high but Spanish-speaking populations are low, educational materials are sparse, especially in isolated communities. Similar gaps are observed in Inner Northern California and the Sierra ecoregions, where moderate to high wildfire risk combines with low Spanish-speaking populations and limited distribution of materials. The Southern California Mountain region shows better targeting of high-risk areas, but outreach remains challenging in its rugged and remote zones, warranting continued attention to education and dissemination. The cluster and regional analyses revealed clear spatial mismatches – regions with large Spanish-speaking population, such as Central Valley and Central Coast, often lacked adequate materials – confirming second hypothesis of unequal geographic distribution.

**Table 3:** Cluster analysis results for different ecoregion

Ecoregion	Clusters present	Wildfire risk	Spanish- speaking population	Material availability	Key observations
Southern California	1,2,3,4	High	High	High	Critical need for ongoing targeted outreach due to high population density and wildfire risk.
Central Valley and Central Coast	1,2,3,4	Moderate to High	High	Limited	Significant gaps in outreach; increased focus required to boost material distribution and effectiveness.
Outer Northern California	2,3	High	Low	Limited	Sparse distribution of materials; increased efforts needed for reaching isolated communities.
Southern California Mountain	1,2,3,4	Moderate to High	Moderate	Moderate	High risk areas well targeted, but outreach gaps remain in remote areas.
Inner Northern California	2,3	Moderate to High	Low	Limited	Gaps in outreach; efforts needed to reach low-population areas at moderate risk.
Sierra Region	2,3	Moderate to High	Sparse	Limited	Educational outreach is sparse; expanded efforts required to mitigate future risks.

### Correspondence Analysis

**Fig. 8:**
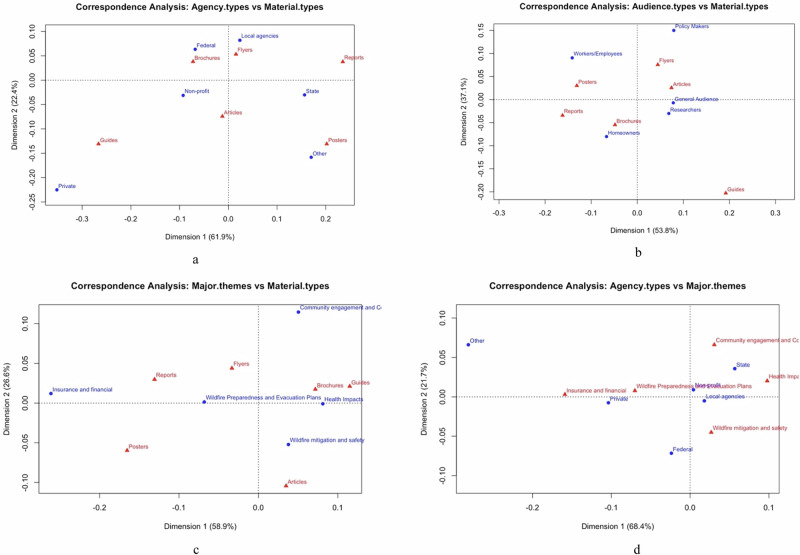
Correspondence analysis (CA) visualizations illustrating relationships among agency types, material types, audience types, and major themes in Spanish-language wildfire materials. **a** Association between agency types and material types, highlighting how different organizations preferentially produce specific formats. **b** Association between audience types and material types, showing how material formats align with intended audiences. **c** Association between major thematic categories and material types, indicating which formats emphasize particular wildfire-related themes. **d** Association between agency types and major themes, revealing how organizational roles correspond to thematic priorities in wildfire communication.

The correspondence analysis (Fig. 8a–d) revealed critical associations among agency types, material types, audience types, and major themes. The axes (Dimension 1 and Dimension 2) represent the two principal dimensions that capture the largest share of variation in how categories are associated with one another. The percentages shown on each axis (e.g., 61.9% or 22.4% (Fig. 8a)) indicate the proportion of total variance explained by that dimension.

Overall, the correspondence plots highlights key associations—for instance, Federal agencies are most strongly associated with “Brochures” and “Flyers,” while state agencies emphasize “Reports” and “Posters.” Local agencies primarily use “Flyers,” whereas non-profits distribute a balanced mix of “Brochures,” “Flyers,” and “Articles.” Audience-specific patterns show that “Workers/Employees” are mainly targeted through “Posters,” “Homeowners” through “Reports” and “Brochures,” the “General Audience” through “Articles,” and “Researchers” through both “Flyers” and “Articles.” Thematic analysis indicates that “Community Engagement and Collaboration” is commonly addressed with “Flyers” and “Brochures,” while “Wildfire Preparedness and Evacuation Plans” are communicated through “Reports,” “Flyers,” and “Brochures.” “Health Impacts” are often presented in “Brochures” and “Guides,” whereas “Wildfire Mitigation and Safety” is typically discussed in “Articles.” Lastly, organizational priorities vary federal agencies focus on “Mitigation and Safety,” private agencies emphasize “Insurance and Financial” themes, and non-profits and local agencies prioritize “Preparedness,” “Community Engagement,” and “Health Impacts.” State agencies also target “Community Engagement” and “Health Impacts,” with some emphasis on “Preparedness.” These findings illuminate the differing strategies and content preferences of various stakeholders engaged in wildfire education outreach across California. The thematic analyses and material type classification showed a dominance of general preparedness content in Spanish-language materials, with limited emphasis on specific issues like smoke exposure and post-disaster recovery, thereby supporting the fourth hypothesis regarding thematic alignment.

## Discussion

Findings provide a comprehensive view of wildfire educational material trends, exposing critical disparities between English and Spanish-language resources, variations in organizational involvement, and uneven regional distribution. Notably, the Central Valley—an area with both moderate to high wildfire risk and a dense Spanish-speaking population—exhibits a severe lack of Spanish-language materials. This gap points to a systemic oversight in disaster planning, echoing environmental justice concerns that highlight inadequate service to low-income and minority communities (Méndez et al. 2020). Although the overall production of materials has increased over time—particularly due to non-profits and local agencies—the inconsistency of Spanish-language material output and limited engagement with specific audiences remain significant challenges. The shortage of culturally and linguistically relevant resources places Latino communities in regions like the Central Valley and Southern California at greater vulnerability. While the Central Valley experiences few wildfires, its low availability of materials underscores a key distinction between modeled wildfire risk and experienced impacts. The development of outreach materials may be driven more by direct and indirect wildfire effects such as smoke exposure and poor air quality than by local burn probability. Communities in Central Valley often face significant indirect risks from nearby fires, suggesting the preparedness communication should address both fire occurrence and secondary impacts that shape public vulnerability.

Efforts on wildfire preparedness, health impacts, and mitigation are increasing, targeted improvements are still needed to ensure Spanish-speaking populations are adequately equipped to face wildfire threats. For instance, CAL FIRE’s “Ready, Set, Go!” guide exemplifies an effective wildfire preparedness resource that clearly communicates evacuation planning, defensible space creation, and household safety measures in both English and Spanish. Similarly, the University of California Cooperative Extension’s “After the Fire” factsheets provide accessible, post-fire recovery guidance tailored to homeowners and agricultural workers. These materials demonstrate the value of clear language, visual accessibility, and cultural relevance in promoting community preparedness and resilience. Highlighting such examples underscores the importance of designing future outreach materials that are not only technically accurate but also socially inclusive and actionable for diverse audiences.

While this study quantifies the production and availability of wildfire-related educational materials, the number of materials produced should not be interpreted as a direct measure of outreach impact or community engagement. Production counts reflect the extent of material development efforts but do not capture how widely this materials are distributed, accessed, or understood by target audiences. The trend analysis of English-language materials reveals an increasing focus on themes such as “Health Impacts,” “Wildfire Mitigation and Safety,” and “Wildfire Preparedness and Evacuation Plans,” aligning with research on climate-driven wildfire intensification and the need for comprehensive public education (Schoennagel et al. 2004; Westerling et al. 2006). However, the limited representation of collaborative and participatory message types in Spanish-language materials highlights a potential barrier. These gaps are particularly relevant in Latino communities, where communal action and family networks are key to effective disaster response. The growing presence of preparedness-oriented Spanish materials supports existing literature on the importance of public education in disaster risk reduction (McCaffrey et al. 2013; Steelman and McCaffrey 2013). Evacuation plans and preparedness messaging, especially in Spanish, play a crucial role in reducing property loss and saving lives during wildfire events (Cohn et al. 2006).

Non-profit and local agencies emerge as critical actors, showing strong positive trends in material production. This is consistent with earlier findings emphasizing the role of grassroots and community-based organizations in fostering culturally appropriate and geographically targeted disaster preparedness (Eisenman et al. 2007; Collins and Bolin 2009). Their importance is reinforced by environmental justice research that shows vulnerable populations often depend on these entities when state and federal outreach falls short (Méndez et al. 2020; Maldonado et al. 2013). Mann–Kendall trend tests (0.805 for non-profits, 0.743 for local agencies) confirm their growing impact, particularly in creating materials tailored to the needs of Spanish-speaking Californians.

Conversely, federal and state agencies show more consistent but less statistically significant involvement. This finding supports a multi-level governance approach in disaster preparedness, where state and federal agencies offer strategic guidance while local organizations implement tailored outreach (McCaffrey and Olsen 2012; Lindell and Perry 2012). Local actors, embedded within communities, understand the cultural, linguistic, and socioeconomic contexts that influence preparedness. Their rising role reflects a shift toward decentralized, context-sensitive wildfire education strategies that enable rapid and relevant dissemination of Spanish-language materials during times of heightened risk.

This study reinforces existing literature by highlighting the prioritization of homeowners and general audiences—especially those in the wildland-urban interface (WUI)—in wildfire outreach materials, consistent with efforts promoting defensible space and evacuation readiness (Cohen 2000; Syphard et al. 2017). However, workers and researchers, particularly those in agriculture and outdoor labor sectors, remain underrepresented despite their exposure to both the direct and indirect impacts of wildfires- such as smoke, heat and disrupted working conditions highlighting there is the need for broader inclusion in outreach and preparedness (Fothergill and Peek 2004). The widespread use of brochures and flyers reflects their proven effectiveness in crisis communication and accessibility, particularly in Spanish-language outreach (Lachlan et al. 2019; Calkin et al. 2015).

Educational and informational message types showed significant growth, supporting the dual role of awareness and action in disaster communication (Westcott et al. 2020; Mileti 1999). However, collaborative and participatory messaging remains limited despite strong support in the literature for shared governance approaches to enhance resilience (Paveglio et al. 2018; Johnson et al. 2014).

The cluster analysis across California’s ecoregions reveals stark disparities in material distribution, risk exposure, and Spanish-speaking population density. The Southern California ecoregion (Fig. 7a), with high wildfire risk and dense populations, demonstrates significant material availability—particularly in WUI zones—but remote areas remain underserved. This mirrors prior findings on the complexity of wildfire management in high-risk urbanized landscapes (Cohen 2000). The Central Valley and Central Coast (Fig. 7b), despite their linguistic and fire-risk profiles, suffer from material gaps—an ongoing issue linked to language barriers and structural inequities (Collins and Bolin 2009; Fothergill and Peek 2004). In Outer Northern California (Fig. 7c), material availability remains low amid high wildfire risk, consistent with literature on the challenges of rural preparedness (Lindell and Perry 2004). Similar patterns are evident in the Inner Northern California and Sierra ecoregions (Fig. 7e–f), which combine high wildfire exposure with limited outreach—an issue exacerbated by rural geography and low-population density (Cutter and Finch 2008). The Southern California Mountain region (Fig. 7d) shows better outreach in high-risk zones but still reveals critical gaps in remote terrain, confirming prior assessments of the difficulty in reaching mountainous communities (Syphard et al. 2017; Keeley and Fotheringham 2001). These patterns reinforce the need for spatially and culturally tailored outreach strategies that reach underserved populations.

The correspondence analysis (Fig. 8a–d) offers a detailed view of stakeholder roles in wildfire education outreach across California, aligning with prior research while revealing new insights. Federal agencies’ use of brochures and flyers reflects their top-down communication strategies (Steelman and McCaffrey 2013; Lachlan et al. 2019), and state agencies’ focus on reports and posters corresponds to their regulatory roles (McCaffrey and Olsen 2012; Calkin et al. 2015). Local agencies rely on flyers, reflecting limited capacity (Eisenman et al. 2009), while non-profits’ balanced use of brochures, flyers, and articles highlights their adaptability and cultural responsiveness (Méndez et al. 2020; Collins and Bolin 2009). Audience-specific targeting aligns with previous findings: homeowners receive detailed materials (Cohen 2000; Syphard et al. 2017), and workers are engaged via posters. Thematically, accessible formats are used for “Community Engagement” and “Preparedness” (Westcott et al. 2020; Paveglio et al. 2018), while “Mitigation and Safety” relies on articles (Moritz et al. 2004; North et al. 2012). A notable contribution is identifying private agencies’ focus on insurance themes, which remains understudied. Despite overall alignment with past literature, underuse of participatory messaging persists, highlighting the need for inclusive strategies to better serve Spanish-speaking and underserved communities (Paveglio et al. 2018; Nielsen-Pincus et al. 2018).

Overall, this study provides a data-driven framework for assessing the alignment between wildfire preparedness resources and community needs across California. By identifying gaps and regional disparities—particularly in Spanish-language outreach—this research lays the groundwork for inclusive, equitable disaster preparedness strategies. As wildfire risk intensifies due to climate change, it is essential to ensure that all communities, especially Spanish-speaking and marginalized groups, receive timely and culturally appropriate preparedness information. Future research should explore the effectiveness of different communication formats and collaborative models in improving disaster readiness across diverse populations.

## Implications and Conclusions

This study of wildfire-related educational materials in California highlights several interconnected policy and practice implications for enhancing wildfire preparedness and addressing disparities in outreach. One of the key areas for improvement is the inconsistent production of Spanish-language materials, which underscores the need for more resources and tailored programs. Policymakers should prioritize investment in translation services, bilingual media campaigns, and partnerships with trusted community organizations in high risk regions. To make collaboration more effective, general recommendations such as increasing collaboration can be anchored in tested strategies – for example, expanding Community Emergency Response Teams (CERT), supporting bilingual Fire Safe Councils, and engaging local schools, agricultural networks to build sustained wildfire awareness and preparedness capacity within Spanish-speaking and underserved communities. In parallel, the crucial role of non-profits and local agencies in bridging gaps, especially in underserved areas, should not be overlooked. Increasing financial support and fostering partnerships with state and federal entities can further amplify their impact. Additionally, while brochures and flyers are effective for quick dissemination, a more balanced approach to material types is necessary. More comprehensive resources, such as reports and guides, should be widely distributed, particularly in high-risk areas, with digital platforms integrated to improve accessibility. Addressing outreach gaps in rural regions, such as Northern California and the Sierra, should also be a priority, with policies focusing on deploying mobile units or information vehicles – for example, traveling vans or trailers that deliver wildfire-related educational materials, improving internet access, and partnering with local leaders to ensure equitable distribution of materials. Furthermore, strengthening wildfire preparedness and evacuation plans by regularly updating and tailoring them to regional risks, in collaboration with local governments and communities, can enhance effectiveness. Increasing collaboration between public agencies, private entities, and communities is another vital step. By incorporating participatory strategies like workshops and local preparedness committees into outreach programs, agencies can foster greater engagement. Resource allocation should also be data-driven, ensuring that high-risk areas, particularly those with large Spanish-speaking populations, receive prioritized support. Expanding digital outreach, through online resources and mobile applications, can further ensure timely dissemination of information, particularly by using geolocation tools and targeted advertising. Finally, continuous monitoring and evaluation of wildfire education programs is essential, with metrics established to assess public engagement, preparedness outcomes, and material availability, ensuring that programs remain effective and adaptive to evolving wildfire risks.

## Data Availability

The datasets are available from the corresponding author upon reasonable request.
